# Gaps in the access to endovascular thrombectomy for acute ischaemic stroke: estimating neurointerventional training needs and modelling implementation impact based on current thrombectomy indicators

**DOI:** 10.1093/esj/aakag026

**Published:** 2026-05-13

**Authors:** Adnan Mujanovic, Michele Romoli, Giacomo Sferruzza, Alice Accorroni, Razvan A Radu, Alicia Gonzalez-Martinez, Mihai R Ionescu, Roland Schwab, Pervinder Bhogal, Alessandro Sodero, Thomas C Truelsen, Jana Midelfart-Hoff, Silke Walter, Melinda B Roaldsen, Francesa R Pezzella, Christian A Taschner, Georgios Tsivgoulis, Nathalie Nasr, Diana A de Sousa, Jan Gralla, Simona Sacco, Urs Fischer, Hanne Christensen

**Affiliations:** Department of Diagnostic and Interventional Neuroradiology, Inselspital University Hospital Bern, University of Bern, Bern, Switzerland; Resident and Research Fellow Section, European Academy of Neurology, Vienna, Austria; Resident and Research Fellow Section, European Academy of Neurology, Vienna, Austria; Department of Neuroscience, Neurology and Stroke Unit, Maurizio Bufalini Hospital, AUSL Romagna, Cesena, Italy; Resident and Research Fellow Section, European Academy of Neurology, Vienna, Austria; Department of Neurology, Vita-Salute San Raffaele University, Milan, Italy; Resident and Research Fellow Section, European Academy of Neurology, Vienna, Austria; Geneva Memory Centre, Division of Geriatrics, Department of Rehabilitation and Geriatrics, Geneva University Hospitals, Geneva 1205, Switzerland; Resident and Research Fellow Section, European Academy of Neurology, Vienna, Austria; Department of Neurology, University Emergency Hospital Bucharest, Bucharest, Romania; Department of Clinical Neurosciences, “Carol Davila” University of Medicine and Pharmacy, Bucharest, Romania; Resident and Research Fellow Section, European Academy of Neurology, Vienna, Austria; Department of Neurology, University Hospital de la Princesa, Madrid, Spain; Department of Neurology, Instituto de Investigación Sanitaria Princesa (IIS-Princesa), Madrid, Spain; Resident and Research Fellow Section, European Academy of Neurology, Vienna, Austria; Department of Neurology, Colentina Clinical Hospital, Bucharest, Romania; University Clinic for Neuroradiology, University Hospital Magdeburg, Magdeburg, Germany; Department of Interventional Neuroradiology, The Royal London Hospital, Barts NHS Trust, London, United Kingdom; Department of Neurology, IRCCS Fondazione Don Carlo Gnocchi, Florence, Italy; Department of Neurology, Copenhagen University Hospital Rigshospitalet, Copenhagen, Denmark; Department of Neurology, Haukeland University Hospital, Bergen, Norway; Department of Neurology, Saarland University Clinic, Homburg, Germany; Department of Clinical Research, University Hospital of North Norway and Department of Clinical Medicine, UiT The Arctic University of Norway, Tromsø, Norway; Stroke Unit, Azienda Ospedaliera San Camillo Forlanini, Roma, Italy; Department of Neuroradiology, Medical Center—University of Freiburg, Faculty of Medicine, University of Freiburg, Freiburg, Germany; Second Department of Neurology, School of Medicine, National and Kapodistrian University of Athens, “Attikon” University Hospital, Athens, Greece; Department of Neurology, Poitiers University Hospital, University of Poitiers, Poitiers, France; Stroke Center, Lisbon Central University Hospital, ULS São José, University of Lisbon, Lisbon, Portugal; Department of Diagnostic and Interventional Neuroradiology, Inselspital University Hospital Bern, University of Bern, Bern, Switzerland; Department of Biotechnological and Applied Clinical Sciences, University of L'Aquila, L'Aquila, Italy; Department of Neurology, Inselspital University Hospital Bern, University of Bern, Bern, Switzerland; Department of Neurology, Bispebjerg Hospital, Copenhagen, Denmark

**Keywords:** access, endovascular therapy, neurointerventionalists, stroke

## Abstract

**Introduction:**

Endovascular therapy (EVT) has become an increasingly important part of acute stroke management. However, the lack of trained neurointerventionalists represents a key barrier in expanding availability of EVT in Europe. This project aimed to investigate the association between the number of neurointerventionalists and overall EVT rates.

**Patients and methods:**

A cross-sectional analysis was conducted using publicly available data from the Global Burden of Disease Report 2021 and the Stroke Action Plan for Europe (Stroke Service Tracker). Data on the number of neurointerventionalists across 35 European countries were surveyed through a structured survey distributed via the Resident and Research Fellow Section (RRFS) of the European Academy of Neurology (EAN). Correlation analyses were performed to estimate the association between neurointerventionalist density per served population, EVT rates and stroke-related mortality and morbidity.

**Results:**

Survey response rate was 71% (25/35 countries). The proportion of acute ischaemic stroke patients treated with EVT ranged from 0.05% to 14.96% of people with ischaemic stroke, and the number of neurointerventionalists ranged from 9 to 137 per country and from 0.3 to 7.5 per million inhabitants. There was a positive correlation between the number of neurointerventionalists per population served and EVT rates (Spearman coefficient ρ = 0.507; 95% CI, 0.209–0.719). Greater availability of trained neurointerventionalists was moderately associated with lower national ischaemic-stroke mortality (ρ = −0.473; 95% CI, −0.746 to −0.065) and lower overall disability-adjusted life years (ρ = −0.444; 95% CI, −0.729 to −0.027).

**Discussion and conclusion:**

The number of neurointerventionalists correlates positively with the annual volume of EVT across European countries; higher EVT rates were also associated with lower stroke-related mortality and disability; however, these associations are unadjusted for other important confounders and causality cannot be inferred. These data suggest an urgent need to increase neurointerventional capacity in Europe, for example, by expanding dedicated national training programmes and enhancing support from national and international professional societies.

## Graphical Abstract

**Graphical Abstract:**
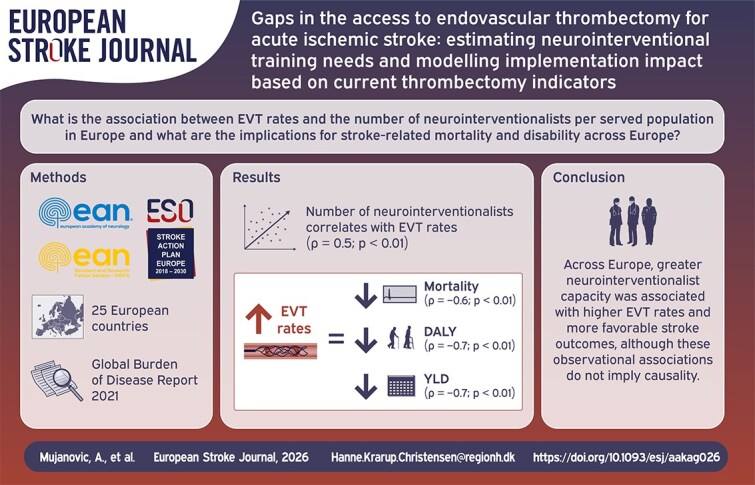

## Introduction

Endovascular therapy (EVT) is considered the first-line treatment for patients presenting with large-vessel occlusion acute ischaemic stroke.^1^^,^^2^ However, there are still areas in Europe with very limited access. A task force has recently reported that the proportion of patients with acute ischaemic stroke treated with EVT in Europe was on average less than 2% in 2016 and less than 7% in 2019.^3^^,^^4^ A survey of Mission Thrombectomy 2020+ global network across 75 countries reports that access to EVT is only 3%, with major disparities between low-, middle- and high-income countries.^5^

As the European population continues to age, the annual number of stroke events is projected to rise.^6–8^ Furthermore, evidence suggests that an increasing number of stroke-patient subgroups could benefit from EVT beyond the criteria defined by current guidelines. These groups include patients with large ischaemic core, patients presenting within a late time window or patients with posterior occlusion strokes.^9^^,^^10^ These findings support the potential expansion of EVT eligibility criteria and indicate that, in the coming years, the capacity for EVT should increase accordingly to meet the population needs.^11^

To address this gap and ensure timely access to EVT for all eligible patients, careful planning regarding funding and workforce allocation is imperative. This should encompass not only physical infrastructure but also on-site multidisciplinary teams equipped to provide comprehensive care for all EVT-eligible acute ischaemic stroke patients across Europe.^7^ A survey of neurology residents revealed that the most common reason for not initiating EVT in eligible stroke patients (in 34 out of 44 European countries) was a lack of on-site neurointerventionalists.^12^ In view of the former considerations, we sought to investigate the association between EVT rates and the number of neurointerventionalists per served population in Europe. Also, we explore the relationship between the availability of neurointerventionalists and morbidity and mortality in patients presenting with acute ischaemic stroke.

## Methods

### Study design

This multicentre cross-sectional study collected data on stroke incidence and on EVT rates from publicly available sources, including the Global Burden of Diseases (GBD) 2021 study and the Stroke Action Plan for Europe (Stroke Service Tracker). Data on the number of neurointerventionalists were obtained through a survey conducted among the national representative network of the Residents and Research Fellow Section (RRFS) at the European Academy of Neurology (EAN), members of the EAN Stroke Panel and the European Stroke Organisation (ESO), as detailed in the sections below. This study was conducted according to the Consensus-Based Checklist for Reporting of Survey Studies (CROSS)^13^ and in accordance with the Declaration of Helsinki.

### Data on stroke incidence and EVT rates

Epidemiological data, including mortality, disability-adjusted life years (DALYs) and years lived with a disability (YLD) for acute ischaemic stroke in the European region, were obtained from the GBD 2021 study (Seattle, United States: Institute for Health Metrics and Evaluation [IHME], 2022; available from https://vizhub.healthdata.org/gbd-results). Countries with less than 100,000 inhabitants (Monaco, Andorra, Vatican City, Liechtenstein and San Marino) were excluded from the evaluation.^14^ Global Burden of Diseases 2021 data are publicly available, and these quantify health loss for 371 diseases in 204 countries and territories, including measures of prevalence, disease severity and death that together constitute a comprehensive assessment of disease burden.^14^ Data for acute ischaemic stroke were cross-referenced for consistency for the years 2020–2022 with the publicly available Eurostat dataset.^15^

Key performance indicators for acute stroke including the total number of stroke units treating acute ischaemic stroke patients with intravenous thrombolysis (IVT), total number of stroke units treating acute ischaemic stroke patients with EVT, rate of acute ischaemic stroke patients treated with IVT, rate of acute ischaemic stroke patients treated with EVT in the European region were acquired through the publicly available dataset of the Stroke Service Tracker.^16^ Stroke Service Tracker is the biggest stroke care improvement project in Europe led by the ESO. The data are gathered by the Stroke Service Tracker National Coordinators who report country-level data (organisation of stroke care, specific pathways, incident strokes, early mortality) into a REDCap database on an annual basis. Only aggregated summary data, such as key performance indicators, stroke care organisation, specific care pathways, incident stroke rates and early mortality were collected. Although Stroke Service Tracker National Coordinators report early mortality (discharge, 30 days and 3 months), these data were not used in the main correlation or regression analyses due to heterogeneity in follow-up windows across countries. For each entry, the data source (eg, mandatory national registry with auditing or estimates) was documented, acknowledging variability in data quality across countries.^16^

### Data on the number of neurointerventionalists

The data on the number of neurointerventionalists, the number of centres offering 24-h EVT service, the average number of work hours per neurointerventionalist per week and the setting in which EVT services were provided across the European region between 2020 and 2024 were surveyed in a structured manner. To develop the survey, we first created a pilot version, which was revised by members of the EAN Stroke Panel management group, the RRFS Office, representatives from the ESO and members of the Stroke Service Tracker, including patient representatives. After incorporating the feedback of this core group, the survey was distributed in 2 waves. The initial survey invitation was sent out on 5 November 2024 through the national representatives network of the RRFS. This network includes national representatives across 35 European countries who are selected by the corresponding National Neurological Society and maintain close ties with them. Two follow-up reminders were sent out to non-responders, with each reminder being sent after a 2-week period. If no RRFS country representative was available, alternative representatives were identified through the members of the EAN Stroke Panel or through the network of ESO.

The survey included 29 questions on 5 separate web pages. The full survey is available in the Online Supplement. The survey questions explored demographic and contact data, the number of practising neurointerventionalists and settings in which EVT was offered. For countries with a large number of EVT centres (eg, Germany, France, Italy) we obtained the number of employed neurointerventionalist per centre on a specific date (1 July of each year) to avoid any temporary variations in employment activities throughout the year. For the purposes of this survey, we defined a neurointerventionalist as a physician formally trained in a neuro-specialty (neurology, neurosurgery or neuroradiology) who performs endovascular procedures for the treatment of acute ischaemic stroke. Given the variability in data collection systems across European countries, the official source for the number of neurointerventionalist was also recorded to ensure transparency and reliability in data reporting (eg, country registry, national society, country-level coordinators, healthcare ministry, personal communications).^13^

### Statistical analysis

Results are reported as median (interquartile range) and *n* (%) unless specified otherwise. For the main analysis, we first imported the full European country-year dataset and excluded any observations with missing values for either the number of trained neurointerventionalists or EVT rates. Two key variables were derived: neurointerventionalist density (number of neurointerventionalists per million population) and ischaemic stroke incidence per million. To assess the overall association between workforce capacity and treatment rates, we calculated the Spearman correlation coefficient (ρ) between EVT rate and neurointerventionalist density across all country-years. Finally, we fitted an ordinary least-squares linear regression model, regressing EVT rate on neurointerventionalist density. Model performance was evaluated via the coefficient of determination and the statistical significance of the slope coefficient, and the fitted relationship was visualised with a scatterplot and superimposed regression line.

For within-country trends, we adjusted the number of neurointerventionalists to take into account the potential growing expertise having an impact on rates of EVT. To account for the increase in procedural experience over time within each country, we created a cumulative “expertise index.”^17^^,^^18^ This index was initialised in the first year as the country’s neurointerventionalist density divided by the maximum first-year density across all countries. In subsequent years, it was incremented by a fixed base learning rate (0.005, β0) plus an additional factor (0.001, β1) proportional to the annual change in the absolute number of neurointerventionalists, with a ceiling of 1.^17^^,^^18^ While procedural volume would ideally be used to quantify experience, country-level procedure counts are not systematically available across Europe. Thus, the “expertise index” represents an exploratory proxy for accumulated experience. Multiplying raw neurointerventionalist density by this index yields an “effective supply” metric, which may provide illustrative insights into potential within-country trends. Both effective supply and EVT rate were then standardised to within-country z-scores. We first assessed the overall (cross-sectional) association between effective supply and EVT rate using Pearson correlation and ordinary least squares regression on pooled country-year data. To capture within-country dynamics, we fitted a first-difference regression predicting year-to-year changes in standardised EVT rate from changes in standardised effective supply, and we also estimated a country fixed-effects linear model to adjust for time-invariant national characteristics. These sensitivity analyses use raw (unadjusted) neurointerventionalist density and are meant to complement the observed associations from the main analyses (see above).

For estimating the gap for neurointerventionalists, population counts were used to calculate neurointerventionalists per million inhabitants. We used 2 scenarios, 1 independent from economic resources and 1 inter-dependent on economic resources. For the latter, we retrieved each country’s gross domestic product per capita (GDP pc, current US $) for that same year from the World Bank via the WDI R package (indicator NY.GDP.PCAP.CD). To estimate the additional workforce required to achieve best-in-class EVT rate, we first identified the maximum observed national EVT rate. We fit a linear regression model of EVT rate on neurointerventionalists density and GDP pc, and for each country solved the fitted equation for the value of neurointerventionalists density that would yield the benchmark EVT rate at its own GDP pc. The difference between this “needed” and the current density gave the gap in neurointerventionalists per million. Finally, we displayed these per-million gaps as a map of Europe to visualise regional needs. For illustrative purposes, we present a hypothetical scenario in which EVT rates reached 15% in all European countries, based on recent ESO recommendations.^4^ This is done with a log-linear regression model fitted with log-transformed stroke mortality as the dependent variable and EVT rate as the independent variable. This analysis is intended to provide contextual, system-level insight into how EVT access aligns with national mortality patterns, rather than to estimate causal effects of EVT on mortality. Relative mortality reductions were calculated and displayed on a country-level. All statistical analyses and visualisations were implemented in R v 4.0.1. (tidyverse, plm, sandwich, lmtest, broom packages and in-house built packages and functions by M.R.).

## Results

The survey response rate was 71% covering 25 out of 35 European countries. All the respondents answered all survey questions. Comparison of available public indicators (GBD 2021) suggested a higher prevalence of acute ischaemic stroke and of stroke-related mortality in non-responding countries, although several non-responding countries with well-established data systems (eg, UK, Germany, Spain) did not follow this pattern (Figure S1). Data were mostly collected through direct communication with country-level stroke coordinators (8/25, 32%), national societies (7/25, 28%) and personal estimates (6/25, 24%). There were no differences in EVT rates between countries reporting from official/verified sources and those reporting from expert estimates (4.7% vs 5.4%). Male respondents constituted 92% (23/25). The majority were residents or early-career specialists in neurology, neuroradiology or neurosurgery. Endovascular therapy was practised only in a public setting in 60% of countries (15/25) (Table 1). Across 25 European countries, the average number of neurointerventionalists in 2020 was 21 (IQR 9–46) and in 2024 it was 28 (IQR 10–90). The reported average work hours per week per neurointerventionalists increased from 2020 (46 h, IQR 40–60) to 2024 (50 h, IQR 44–60). Summary of survey responses for the period 2020–2024 is shown in Table 1.

**Table 1:** Respondents’ baseline characteristics.

Survey questions and responses	2020	2021	2022	2023	2024
***n* (%)**	25 (100)	25 (100)	25 (100)	25 (100)	25 (100)
**EVT setting (%)**					
** Only in public setting**	15 (60)	15 (60)	15 (60)	15 (60)	15 (60)
** Mostly in public setting**	6 (24)	6 (24)	6 (24)	6 (24)	6 (24)
** Both, with a higher tendency to be done in a public setting**	3 (12)	3 (12)	3 (12)	3 (12)	3 (12)
** Both, with equal tendency to be done in both settings**	–	–	–	–	–
** Both, with a higher tendency to be done in a private setting**	1 (4)	1 (4)	1 (4)	1 (4)	1 (4)
** Mostly in private setting**	–	–	–	–	–
** Only in private setting**	–	–	–	–	–
**Methods of data collection**					
** Country registry**	1 (4)	1 (4)	–	–	–
** National society**	7 (28)	7 (28)	7 (28)	7 (28)	7 (28)
** Healthcare ministry**	2 (8)	1 (4)	2 (8)	3 (12)	2 (8)
** Healthcare board**	–	–	–	–	–
** Direct communication with country coordinator**	8 (32)	9 (36)	9 (36)	8 (32)	8 (32)
** Personal estimates**	6 (24)	6 (24)	6 (24)	6 (24)	7 (28)
** Both country registry and direct communications**	1 (4)	1 (4)	1 (4)	1 (4)	1 (4)
** Other**	–	–	–	–	–
**Number of neurointerventionalists**					
** Number of practising neurointerventionalists (per country)**	21 [9–46]	20 [9–62]	21 [10–70]	25 [10–90]	28 [10–90]
** Average number of work hours per single neurointerventionalist**	46 [40–60]	46 [40–60]	46 [40–60]	48 [44–60]	50 [44–60]
** Number of centres with 24-h-available EVT service (per country)**	5 [3–15]	5 [3–20]	5 [3–20]	7 [4–25]	7 [4–27]

Abbreviation: EVT, endovascular thrombectomy.

Data are displayed as % (*n*), and median [IQR].

**Figure 1:**
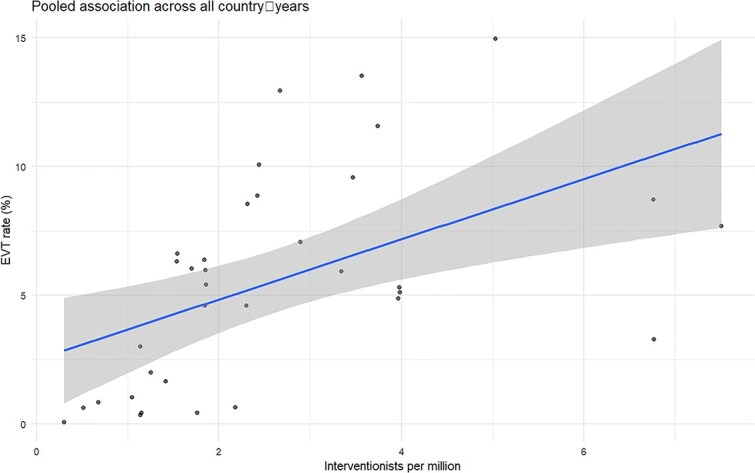
Correlation plot between rate of EVT and reported neurointerventionalists per million inhabitants. Abbreviation: EVT = endovascular thrombectomy.

The proportion of acute ischaemic stroke patients treated with EVT ranged from 0.05% to 14.96% of people with ischaemic stroke, and the number of neurointerventionalists ranged from 9 to 137 per country, or from 0.3 to 7.5 per million inhabitants. Broader ranges across all countries are reported in Table S1. The neurointerventionalist density (number of neurointerventionalists per million population) was moderately and positively associated with national EVT rates. A Spearman correlation yielded ρ = 0.507 (95% CI, 0.209–0.719; *P* = .0019; Figure 1) shows that countries with higher neurointerventionalist density tended to have higher EVT rates. In the unadjusted linear regression, each additional neurointerventionalist per million was associated with a 1.17 percentage-point increase in EVT rate (β = 1.1695; *P* = .0019). The model intercept was 2.50 (SE = 1.09; *P* = .0286), and the overall fit was modest (*R*^2^ = 0.257).

Spearman rank-order correlations showed that greater availability of neurointerventionalists was moderately associated with lower national ischaemic-stroke mortality (ρ = −0.473; 95% CI, −0.746 to −0.065; *P* = .026; Table 2) and lower overall DALYs (ρ = −0.444; 95% CI, −0.729 to −0.027; *P* = .039), but was not significantly related to YLD alone (ρ = −0.228; 95% CI, −0.593 to 0.214; *P* = .308; Figure S2). In contrast, higher EVT rates were strongly and inversely correlated with all 3 outcomes: mortality (ρ = −0.688; 95% CI, −0.860 to −0.375; *P* = .0004), DALYs (ρ = −0.713; 95% CI, −0.872 to −0.417; *P* = .0002) and YLD (ρ = −0.684; 95% CI, −0.858 to −0.368; *P* = .0005; Figure S2).

**Table 2:** Spearman correlation coefficients for neurointerventionalist density and outcomes.

Variable	Outcome	Correlation (95% CI)	P-value
**Neurointerventionalists per million**	Mortality	−0.473 (−0.746 to −0.065)	.026
**Neurointerventionalists per million**	DALYs	−0.444 (−0.729 to −0.027)	.039
**Neurointerventionalists per million**	YLD	−0.228 (−0.593 to 0.214)	.308
**Rate of EVT**	Mortality	−0.688 (−0.860 to −0.375)	.0004
**Rate of EVT**	DALYs	−0.713 (−0.872 to −0.417)	.0002
**Rate of EVT**	YLD	−0.684 (−0.858 to −0.368)	.0005

Abbreviations: DALYs, disability-adjusted life years; EVT, endovascular thrombectomy; YLD, years lived with a disability.

For the regression analysis, only countries with complete data on both neurointerventionalist workforce and EVT rates for all 3 years, 2020–2022, were included, resulting in 11 eligible countries. There was a frequent overall increase in expertise-adjusted supply z-scores and a gain in EVT-rate z-scores (Figure S3). A pooled first-difference regression (Δ*z*_rate ∼ Δ*z*_eff) yields a slope of approximately 0.8 (*P* < .01), meaning that for each 1 SD increase in effective supply, a country can expect about a 0.8 SD rise in its EVT rates. Denmark, Estonia, Finland and Greece showed a transient dip or plateau in 2021 (potentially related to external factors, eg, COVID-19 pandemic), but by 2022 their EVT rates returned to the positive trajectory set by growing supply.

### Neurointerventionalist gap

**Figure 2:**
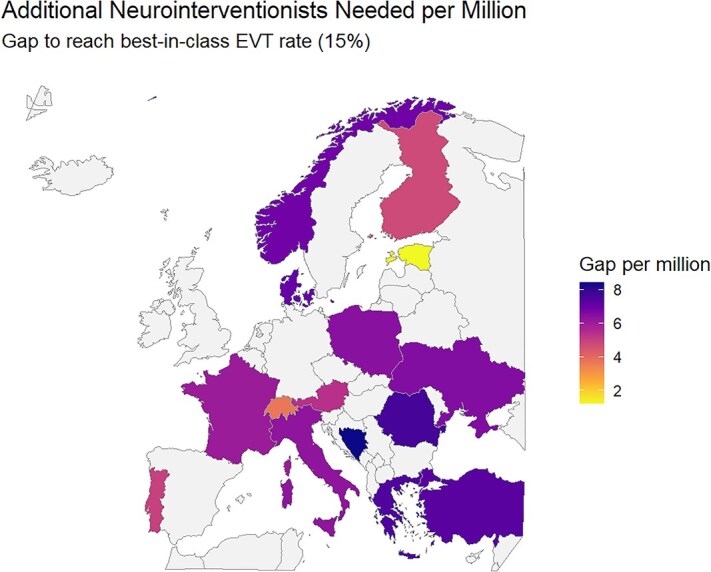
Estimated gap in neurointerventionalists per million inhabitants to reach the best-in-class rate of endovascular therapy (optimal scenario). Only countries with complete data across all variables for the years 2020–2022 were plotted. No countries were selected based on geographic, economic or clinical characteristics. Abbreviation: EVT = endovascular thrombectomy.

**Figure 3:**
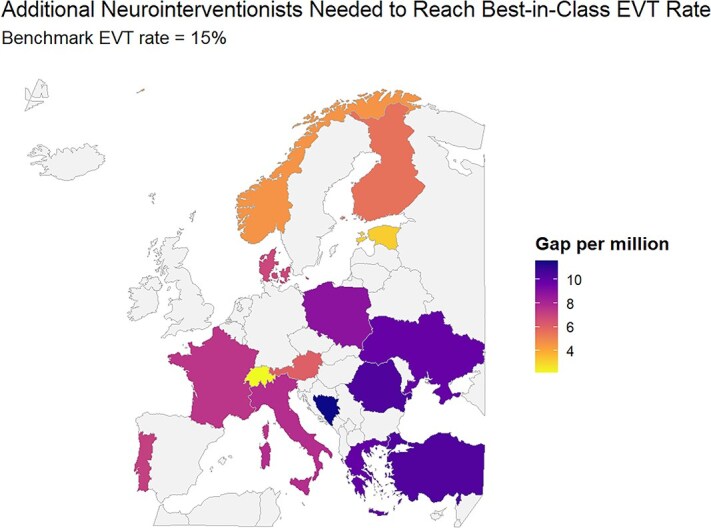
Estimated gap in neurointerventionalists per million inhabitants to reach the best-in-class rate of endovascular therapy, controlling for economic factors (optimal benchmark scenario controlled for gross domestic product per capita). Only countries with complete data across all variables for the years 2020–2022 were plotted. No countries were selected based on geographic, economic or clinical characteristics. Abbreviation: EVT = endovascular thrombectomy.

Across 15 European countries with complete data available for at least 1 year between 2020 and 2022, neurointerventionalist density in 2020–2022 ranged from about 0.3 to 6.8 per million inhabitants. Countries were included only if information on both neurointerventionalist numbers and EVT rates was available for all 3 years. Under the GDP-adjusted linear model, needed densities to achieve the highest observed EVT rate (≈15%) span roughly 6–12 neurointerventionalists per million (Table S1). The resulting per-million gaps translate into headcount increases from only a few neurointerventionalists (eg, Switzerland: +19) up to several hundred (eg, Turkey: +856). Middle-income countries (eg, Ukraine, Bosnia and Herzegovina) require proportionally larger workforce expansions, reflecting both their low baseline EVT rates and economic constraints captured by GDP per capita (Figures 2 and 3).

**Figure 4:**
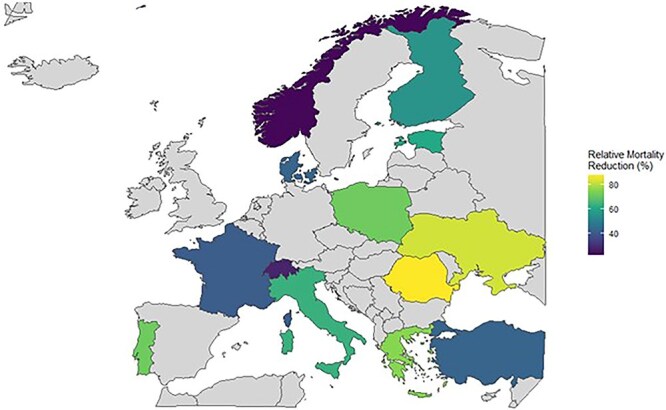
Estimated mortality rate reduction based on ideal scenario of reaching an EVT rate of 15% in all countries, at once. Projections are based on log-linear regression fitted with log-transformed stroke mortality as dependent variable for EVT rate. Only countries with complete data across all variables for the years 2020–2022 were plotted. No countries were selected based on geographic, economic or clinical characteristics. Abbreviation: EVT = endovascular thrombectomy.

In this illustrative scenario, assuming all countries reached a 15% EVT rate and that observed country-level associations remained stable, countries with currently low EVT uptake showed the greatest relative alignment between higher EVT access and lower stroke-related mortality, particularly in Eastern and Southeastern Europe (Figure 4).

## Discussion

This study has the following main findings^1^: Mapping trends from 2020 to 2024 revealed an increase in the number of neurointerventionalists, along with a rise in the number of weekly working hours per individual neurointerventionalist.^2^ Spatial and temporal mapping showed a positive correlation between the number of neurointerventionalists and the annual number of EVT performed per country.^3^ A year-by-year comparison demonstrated a correlation between higher rates of EVT and reductions in stroke-related mortality and post-stroke disability. It is, however, also highly likely that higher numbers of neurointerventionalists and higher rates of EVT are characteristics of well-organised and matured healthcare systems and that the benefits associated with higher numbers of neurointerventionalists also reflect system level robustness.

Prior European-level studies discuss treatment rates and accessibility for acute ischaemic stroke^3^^,^^4^; however, limited data are available for the number of neurointerventionalists. Here we present associations between increases in effective neurointerventionalist work force and EVT. This suggests that workforce availability is one of the factors that may contribute meaningfully to differences in treatment access. This is also in line with prior European surveys showing that regions with higher procedural capacity and more structured on-call networks achieve greater EVT coverage.^3^^,^^4^ The expertise index was developed to provide an illustrative, assumption-based perspective on how accumulated procedural experience might influence EVT rates at the country level. While results suggest a positive association between expertise-adjusted supply and EVT rates, these analyses are exploratory and hypothesis-generating. Importantly, the primary conclusions do not rely on this assumption-driven metric.

The observed temporal increase in neurointerventionalist numbers parallels recent reports of expanding thrombectomy networks in several European regions.^19–21^ However, the association between neurointerventionalist density and EVT rates is unlikely to be strictly linear. At higher workforce densities, EVT rates may plateau due to population size or organisational constraints, and procedural volume per operator may decline, potentially affecting efficiency, quality of care and cost-effectiveness. As most European countries in our dataset remain below these saturation thresholds,^4^ our findings primarily reflect capacity-limited systems rather than supporting unlimited workforce expansion.

Present analysis also extend previous work by linking workforce capacity and health impact across Europe.^19–21^ However, this growth has not been uniform and remains insufficient to meet the rising demand for EVT. In a sensitivity analysis, non-responding countries showed variability in stroke burden and data system quality. While some non-responding countries had limited data systems, others (eg, UK, Germany, Spain) maintain robust national stroke registries. Therefore, the observed workforce and treatment gaps may reflect both true differences in capacity and differences in data availability, rather than uniformly higher stroke burden in non-responding countries. The exploratory analysis on observed associations between higher EVT rates and lower stroke-related mortality and disability are consistent with large-scale population data,^5^^,^^7^ but do not establish causal relationships. Country-level mortality reflects multiple interrelated factors and it should be noted that these associations were not adjusted for other potential confounders, including population health characteristics, comorbidities, pre-hospital care, making these results hypothesis-generating rather than evidence of direct causality.

Likewise, the present study focuses on the availability of neurointerventionalists; however, it is important to recognise that successful EVT service depends on a multidisciplinary effort. Nursing staff, anaesthesiology support, institutional collaborations and adequate imaging and angiography infrastructure are all critical components. Some countries achieve high EVT rates through centralised, high-volume centres with efficient referral pathways, while others rely on more decentralised access models.^22^ Countries such as Denmark, the Netherlands and Switzerland achieve high EVT rates with centralised, high-volume centres and well-coordinated referral networks, whereas countries like Italy and Spain rely on more decentralised EVT provision. Our analyses suggest that, beyond sheer neurointerventionalist density, system organisation, referral efficiency and resource allocation jointly influence EVT delivery. Such differences may explain why some countries achieve higher EVT rates despite comparable workforce numbers. In addition, the costs of EVT procedures and endovascular devices can pose important barriers to access, and addressing these economic considerations is likely to facilitate broader adoption of EVT across different healthcare systems.^23^ In parallel, as EVT represents the final interventional step in the acute stroke pathway, it should be integrated within a comprehensive stroke care network that includes stroke unit management, early rehabilitation and secondary prevention to achieve optimal patient outcomes. These findings should therefore be interpreted as reflecting the potential contribution of neurointerventionalist availability within the broader context of service organisation, resource allocation and economic constraints. Present study also supports efforts to harmonise data collection across Europe and may help inform future initiatives to strengthen acute stroke care.

To address the lack of neurointerventionalists, an international multi-society consensus has already proposed training guidelines for endovascular stroke management, including physicians irrespective of their primary area of residency training (radiology, neurosurgery or neurology).^24^ The reasons why this is most effectively achieved among physicians with a background in the neuroscience is that they possess the pre-requisite clinical, anatomical and pathophysiological understanding required for safe and effective EVT performance.^25–28^ This also aligns with previous reports suggesting that neurointervention develops most effectively in environments where multiple neuroscience specialties collaborate.^25–28^ While addressing workforce shortages is important, expanding EVT practice beyond these core neurodisciplines should be approached with caution. In regions where dedicated neurointerventional personnel are scarce, procedural expertise may be supplemented by cardiologists or peripheral interventionalists. However, their procedural expertise in non-cerebral vascular territories does not necessarily translate into the neurovascular decision-making. Therefore, it remains crucial to maintain procedural safety, quality of care and alignment with established standards of neurovascular practice.

There is rising interest in the field of EVT among neurology residents in Europe who feel they are not sufficiently exposed to neuroimaging nor neurointerventions during their residency training programmes.^12^^,^^26^ Across 27 European countries, 50% of neurology residents have expressed their willingness to apply and undergo further training on EVT, if given the opportunity to do so.^12^ This survey reports intent rather than confirmed enrolment. Actual participation may be influenced by logistical, institutional or country-specific factors. This is mostly due to limited availability of structured neurointerventional training pathways for neurologists and institutional barriers that may restrict access to procedural training slots.^20^^,^^21^ At the same time, the increasing demand for EVT has led to higher workloads per individual neurointerventionalist, which raises concerns about burnout and professional satisfaction within the field.^29^ The current training infrastructure in Europe for provision of acute EVT is insufficient for ensuring that stroke patients will have timely access to first-line stroke therapy.^5–7^ To improve the availability and to close the gap will require expanding training opportunities and potentially including other neurospecialists.^25^^,^^30^

The question of whether future operators should focus exclusively on AIS-related endovascular procedures or be trained across the broader neurointerventional spectrum is context-dependent. In most centres, high-quality EVT requires consistent procedural exposure, rapid decision-making and the ability to manage complex complications such as vessel perforation or dissection requiring stenting.^1^^,^^2^ These skills are best maintained when neurointervention is practised as a primary professional role rather than a part-time activity alongside unrelated clinical duties.^21^^,^^30^ Therefore, neurologists, neurosurgeons and neuroradiologists with appropriate training seem best positioned to serve as full-time neurointerventionalists.^25^^,^^28^ However, hybrid or shared models may be appropriate in low-volume or remote settings in which full-time neurointerventionalist staffing is not feasible. Ultimately, the optimal staffing model depends on centre volume, local stroke incidence and available infrastructure.

## Limitations

Our study is not exempt from limitations. First, methodological differences in data collection exist across the participating countries. To mitigate these variations, information on the specific data sources used in each country was also gathered.^13^ In cases where high-quality data were lacking, the most reliable source was sought through direct contact with the national stroke network representatives or from other stroke units within the country. In some countries, workforce numbers were based on expert estimates rather than centralised registries; however, exploratory analyses comparing countries with different reporting sources showed no differences. The reported increase in weekly working hours per neurointerventionalist is self-reported and should be interpreted cautiously. As a further limitation, the nature of available data forced us to apply a frequentist approach. Although GBD mortality estimates are derived using Bayesian models, they are published as point estimates with uncertainty intervals. A fully Bayesian correlation approach would require access to posterior draws, which are not publicly available. Therefore, we used a frequentist method based on point estimates, as a pragmatic and reproducible approach given current data access constraints. This analysis focuses on the rates of EVT and the number of neurointerventionalists during the same time period. It does not account for other quality performance measures, such as patient selection criteria or time metrics for reperfusion treatment delivery. Similarly, the present study does not account for other resource constraints, such as neuroradiological technicians, nursing staff and angiosuite availability, all of which may impact treatment capacity. In addition, the costs associated with EVT procedures and endovascular devices may limit accessibility, particularly in resource-constrained settings. Present analysis focused on system-level capacity and did not account for centre volumes or spatial accessibility. A further limitation is the absence of data from several European countries due to incomplete or non-standardised reporting of neurointerventional workforce and annual EVT volumes, reflecting the current level (or lack) of data-sharing and cooperation across Europe. Future studies incorporating standardised and comprehensive data across Europe will enable a fully representative assessment of workforce gaps and treatment accessibility. However, this study gathers estimations from 25 countries over Europe providing a general overview of the topic.

## Conclusions

This study found that the number of neurointerventionalists is strongly correlated with the number of EVT per country per year. Higher EVT rates were also associated with lower stroke-related mortality and disability, although, these associations are unadjusted for other important confounders and causality cannot be inferred. These data support the need for further development of educational programmes to increase neurointerventionalist training, supported by appropriate national training programmes as well as national and international professional societies.

### R‌RFS EAN-ESO-SAPE consortium

Simon Fandler-Höfler, Valentino Racki, Sven Zupanic, Rafaela Louka, Kadri-Hebo Kukumägi, Henri Hokkanen, Alexander Balcerac, Konstantinos Melanis, Pablo Antonio Fritz Ruenes, Cecilie Nome, Pawel Wrona, Mafalda Soares, Miguel Serodio, Dmitar Vlahovic, Guillermo Cervera-Ygual, Elena Zapata Arriaza, Ezgi Yilmaz, Margarita Grneva, Giorgi Matchavariani, Katarzyna Krzywicka, Ales Tomek, Marija Bender, Gojko Bogdan, Andreas Hjelm Brandt, Fee Keil, Daniel P.O. Kaiser, Mariam Koka, Dmytro Hrynykha, Manuel Raquena, Marta Olive-Gadea, Danilo Toni, Mauro Silvestrini, Sandra Bracoo, Lorenzo Monasta.

## Supplementary Material

Online_suplement_Tables_And_Figures_aakag026

## Data Availability

Data are available from the corresponding author upon presented research plan proposal.
