# Supplementary material for: Gaps in the access to endovascular thrombectomy for acute ischaemic stroke: estimating neurointerventional training needs and modelling implementation impact based on current thrombectomy indicators
Source: Eur Stroke J. 2026 May 13;11(5):aakag026. doi: 10.1093/esj/aakag026 (PMC13171038; doi:10.1093/esj/aakag026)
Supplement: Online_suplement_Tables_And_Figures_aakag026 [file online_suplement_tables_and_figures_aakag026.pdf]

ONLINE SUPPLEMENT

Gaps in the access to endovascular thrombectomy for acute ischemic stroke: estimating neurointerventional training needs and modelling implementation impact based on current thrombectomy indicators

Table of Contents

- Table S1 Neurointerventionalist gap across countries (Gross Domestic Product per capita adjusted model)
- Figure S1 Ischemic stroke prevalence and stroke-related mortality across European countries
- Figure S2 Correlation between neurointerventionalists density and EVT rates with mortality, DALY and YLD
- Figure S3 Within-country trends in availability of neurointerventionalists and endovascular thrombectomy rates, standardized per potential incremental experience over time.

Table S1 Neurointerventionalist gap across countries (Gross Domestic Product per capita adjusted model)

**Country**	**Year**	**Population**	**EVT rate**	**GDP**	**NI per million**	**NI needed per million**	**Gap per million**
Austria	2022	8978929	5.92	52177	3.34	9.41	6.07
Bosnia and Herzegovina	2020	3280815	0.05	6130.3	0.3	11.85	11.54
Denmark	2022	5873420	6.04	68091	1.7	8.57	6.86
Estonia	2022	1331796	7.68	28451	7.51	10.67	3.16
Finland	2022	5548241	4.87	50438	3.97	9.5	5.54
France	2020	67473651	12.95	39170	2.67	10.1	7.43
Greece	2022	10389971	2	20972	1.25	11.06	9.81
Italy	2023	58997201	8.87	39003	2.42	10.11	7.68
Norway	2022	5434319	6.37	108798	1.84	6.41	4.57
Poland	2022	36889761	4.6	18891	2.3	11.17	8.87
Portugal	2022	10421117	11.56	24621	3.74	10.87	7.13
Romania	2022	19042455	1.02	15558	1.05	11.35	10.3
Switzerland	2022	8740472	14.96	93246	5.03	7.24	2.2
Turkey	2020	83154997	1.64	8638.7	1.42	11.72	10.3
Ukraine	2022	36744634	0.63	4199.7	2.18	11.95	9.77

GDP: Gross Domestic Product , EVT: endovascular thrombectomy , NI: Neurointerventionalist.

Figure S1 Ischemic stroke prevalence and stroke-related mortality across European countries

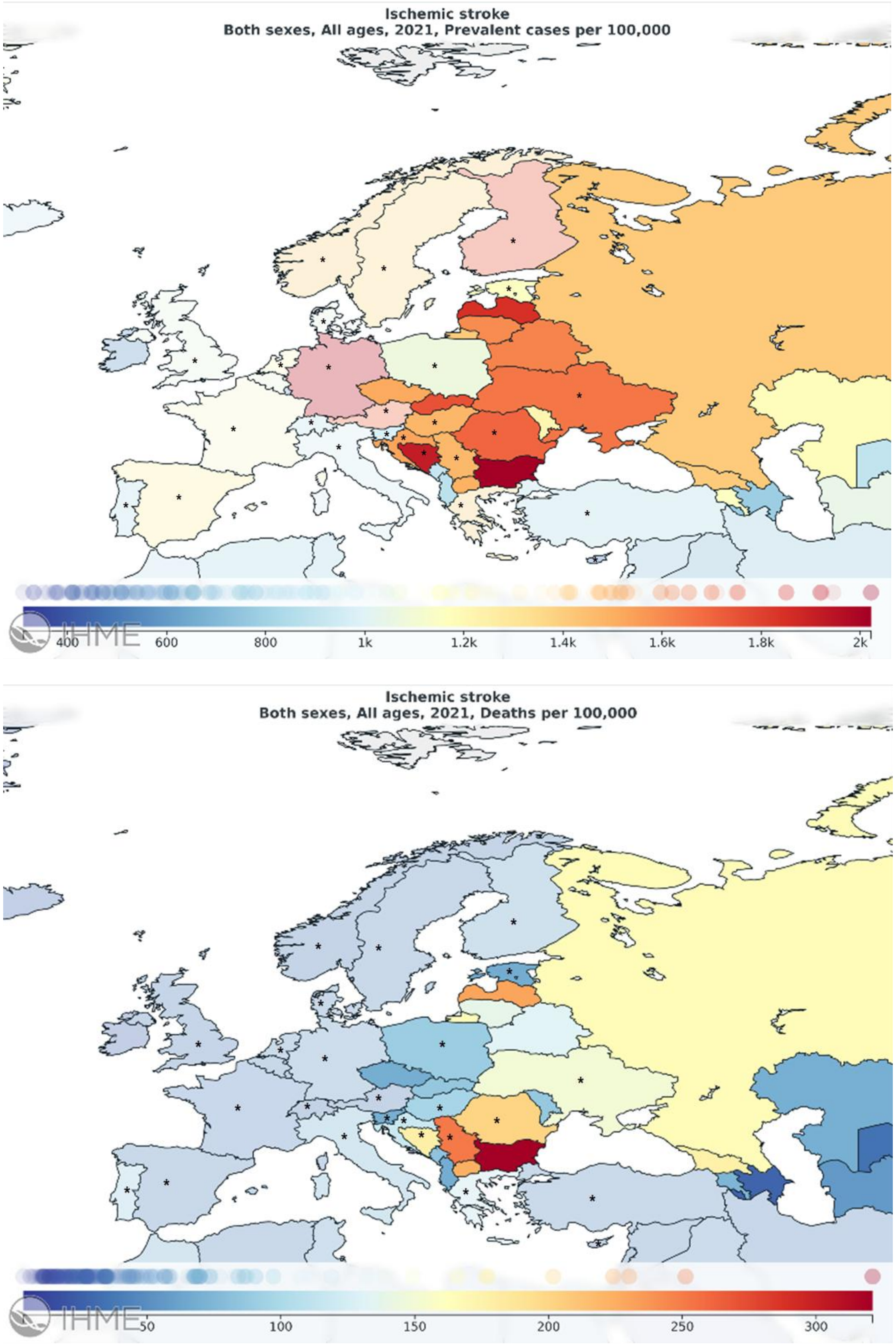

(Top row) Estimated ischemic stroke prevalence per 100,000 population (both sexes, all ages) across European countries according to the Global Burden of Disease (GBD) 2021 dataset. (Bottom row) Estimated stroke-related mortality per 100,000 population (both sexes, all ages) for the same countries. Data source: Global Burden of Disease Study 2021, Institute for Health Metrics and Evaluation (IHME). Countries marked with an asterisk (*) indicates countries that responded to the neurointerventionalist survey.

Figure S2 Correlation between neurointerventionalists density and EVT rates with mortality, DALY and YLD.

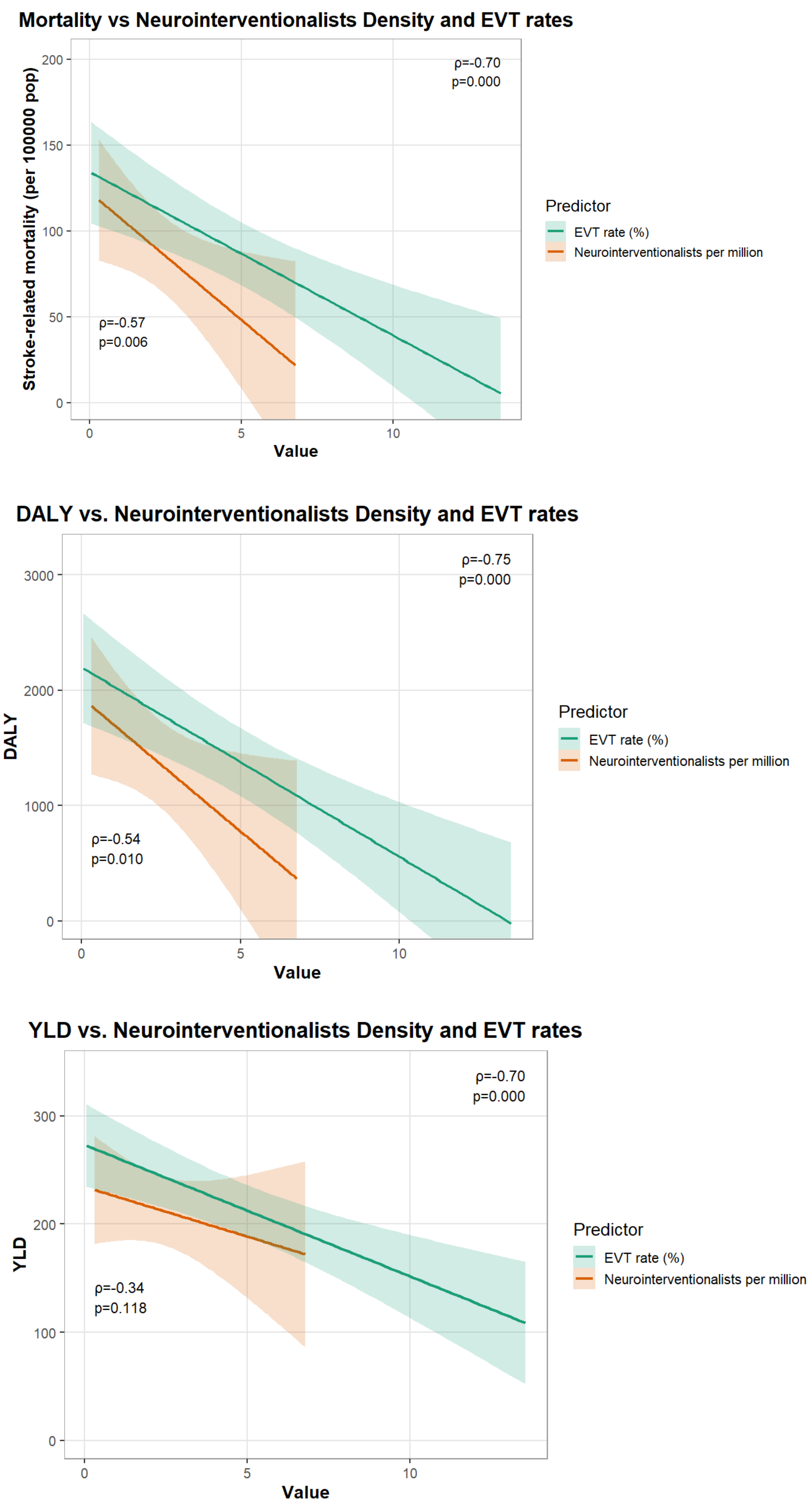

DALY: disability-adjusted life year, EVT (MT): endovascular thrombectomy.YLD: years lived with a disability. Plots illustrate the direction and approximate strength of ecological associations, not absolute predictions

Figure S3 Within-country trends in availability of neurointerventionalists and endovascular thrombectomy rates, standardized per potential incremental experience over time.

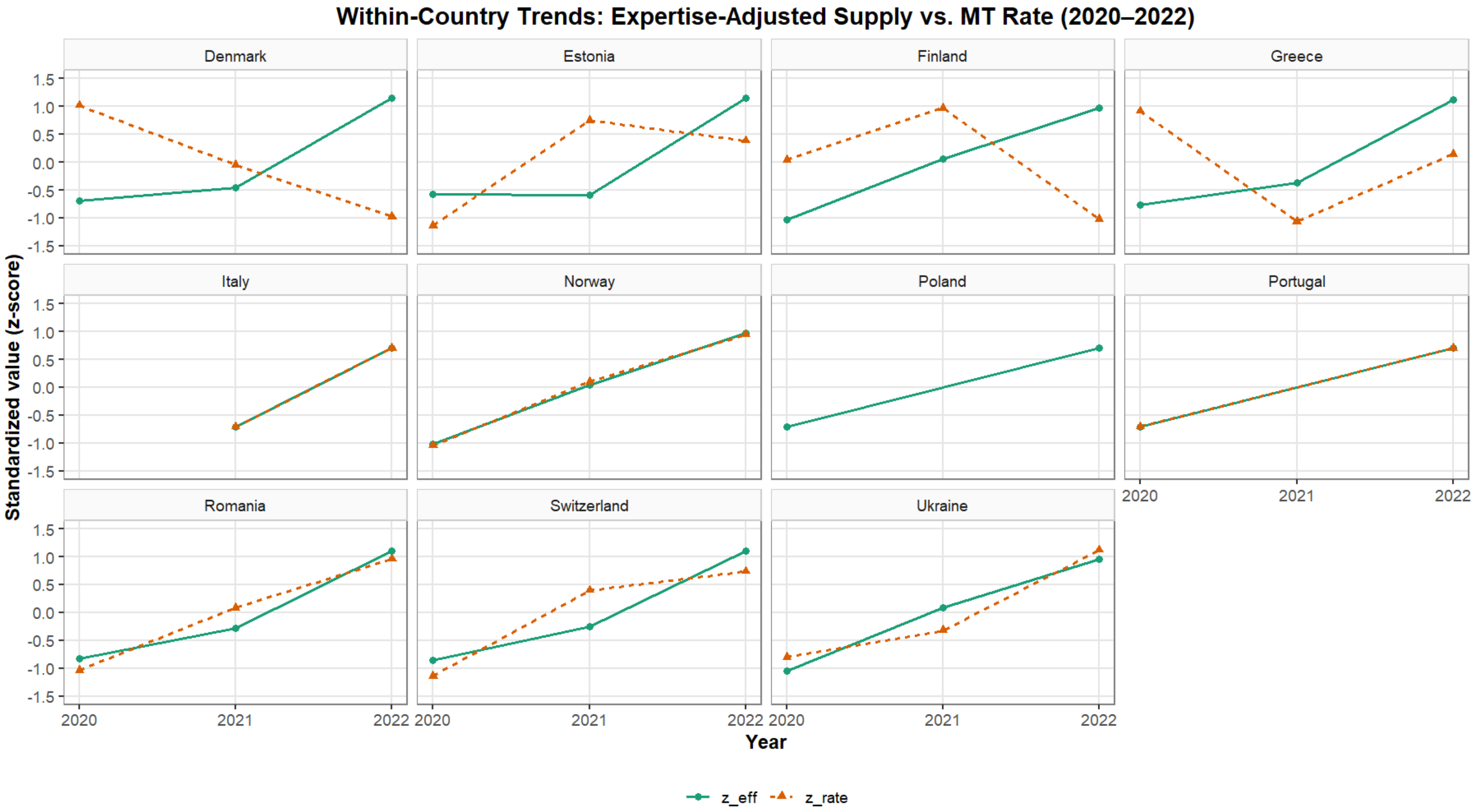

EVT (MT): endovascular thrombectomy
